# Isolated Fallopian Tube Torsion: A Rare Entity

**DOI:** 10.1155/2021/3872201

**Published:** 2021-11-30

**Authors:** Indranil Banerjee, Yatin Thakur, Gargi Mukherjee, Jitendra Jadhav, Amita Sahare

**Affiliations:** Basildon & Thurrock University Hospital, Basildon, Essex, UK

## Abstract

Isolated fallopian tube torsion is an extremely rare occurrence in a young female. The lady concerned presented with acute abdominal pain and the ovaries were normal on the scan with dilated fallopian tubes. On laparoscopy, it was revealed that she was suffering from fallopian tube torsion and laparoscopic salpingectomy was performed. The patient recovered well postoperatively.

## 1. Background

Isolated torsion of the fallopian tube is an extremely rare occurrence in a female patient presenting with acute lower abdominal pain. It does not have any classical signs and symptoms which would prompt an accurate diagnosis. The diagnosis usually comes late and is usually confirmed only on laparoscopy where the gangrenous tube can rarely be salvaged. Strikingly, the ovary can be salvaged in maximum number of cases.

## 2. Case Presentation

The lady concerned was a 22-year-old girl who presented in A&E department with presentation of pain in the left iliac fossa for 5 days. The pain was throbbing in nature. She reported an intensity of about 8/10. There was no association with any bladder or bowel symptoms. There was no previous significant medical or surgical history.

## 3. Examination and Investigation

On examination, she was tachycardic with a pulse rate of 120 bpm. She was normotensive and apyrexic. On examination, she was found to be tender in the region of left iliac fossa. The uterus was found to have a normal size with reduced mobility. There was a lump palpable on the left adnexa on per vaginal examination.

Her blood investigations revealed that she was suffering from anemia (Hb—95 gm/dl), increased white cell count, and increased CRP.

**Figure 1:**
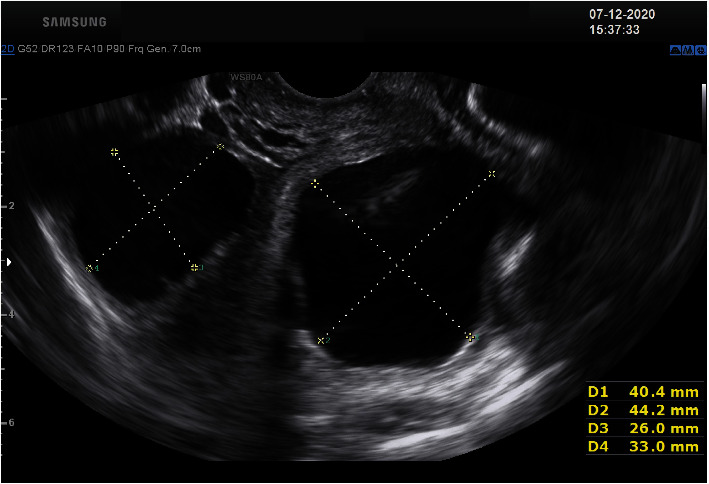
Dilated Tubes on the left side.

**Figure 2:**
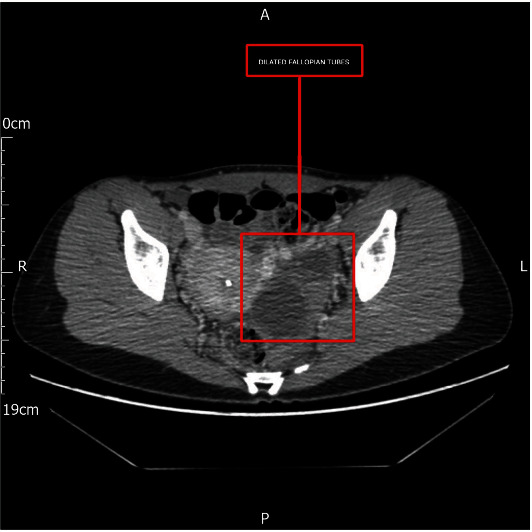
CT scan images. CT scan impression: −7 × 8 cm cystic structure noted in the left adnexa—tubo-ovarian abscess hydrosalpinx.

**Figure 3:**
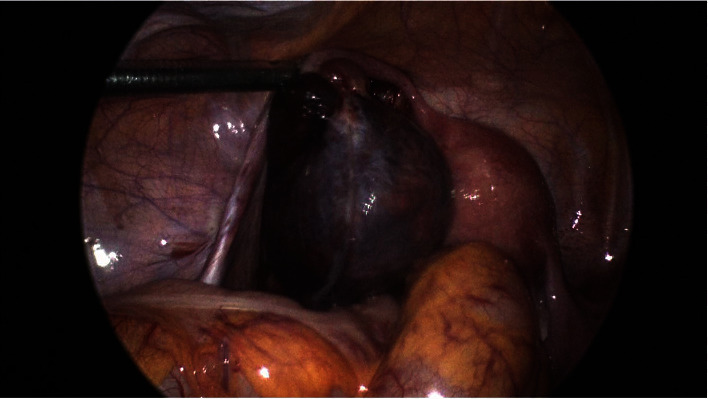
Dilated and gangrenous left fallopian tube.

**Figure 4:**
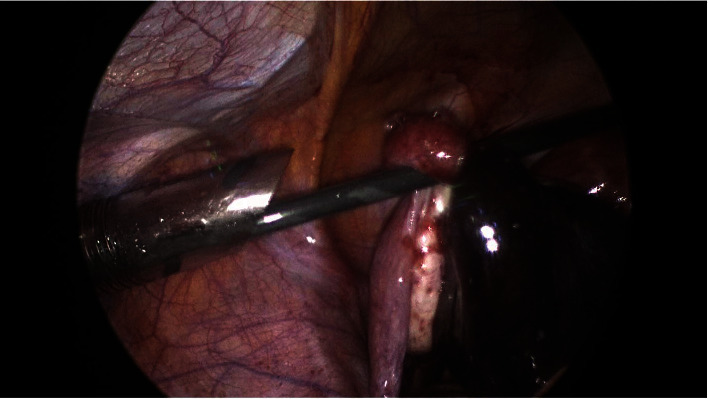
Torted fallopian tube with ipsilateral normal ovary.

**Figure 5:**
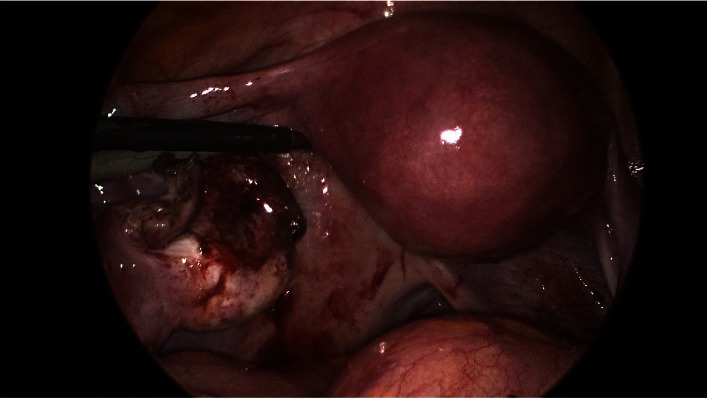
Postresection ipsilateral unaffected ovary.

On imaging, there was a lump seen on the left adnexa (separated from the ovary) on both transvaginal ultrasound and CT scan of lower abdomen on pelvis. Figures 1–5 are shown below.

### 3.1. Ultrasound Images

#### 3.1.1. Report

The uterus was found to be anteverted and anteflexed measuring 9.3 cm × 6 cm × 4 cm. Both ovaries appeared morphologically normal. Right sided fallopian tubes appear normal. Left sided fallopian tube appears dilated—2 × cystic structure noted around the tube measuring 4 cm × 4.4 cm and 2.6 cm × 3.3 cm. There is no free fluid in the pouch of Douglas. Extreme probe tenderness was noted on the left adnexa. Provisional diagnosis: hydrosalpinx—left sided tubo ovarian abscess.

### 3.2. Treatment

On provisional diagnosis of tubo-ovarian abscess, she was consented for laparoscopy ± proceed. On laparoscopy, the left fallopian tube was found to be gangrenous and edematous due to complete torsion. Left sided complete salpingectomy was performed on the same sitting. She was shifted to the ward after the operation, and she was discharged on the next day. Her postoperative first follow-up was scheduled after 7 days, and her postoperative follow-up was uneventful.

### 3.3. Histopathology

On microscopy, there is dilating tubular specimen presumably the fallopian tube which has lost its mucous, and plicae show extensive uniform interstitial haemorrhage probably due to torsion, most likely in keeping with clinical history of torsion. There is no chorionic villous or dense fibrosis or decidua to suggest ectopic gestation.

Diagnosis: left fallopian tube—torsion with haemorrhage.

## 4. Laparoscopy Findings

### 4.1. Differential Diagnosis

Ovarian torsion and acute appendicitis are the close differential diagnosis which might put the diagnosis in dilemma.

### 4.2. Outcome and Follow-Up

The patient recovered uneventfully in the next postoperative day. The lady was reviewed at 6-week follow-up, and she did not have any specific complaint.

### 4.3. Learning Point

- Fallopian tube torsion is a rare but known etiology of acute abdominal pain in a young women
- The ovaries might appear normal on ultrasound and CT scan, but the entire clinical picture must be taken into account before reaching a diagnosis
- The recommended management is laparoscopic fallopian tube detorsion if the tube is still viable. If gangrenous, laparoscopic salpingectomy is the preferred option

## 5. Patient's Perspective

*Though it makes us deeply concerned that our daughter has lost one of her tubes at a so young age, yet we understand that as there was a risk of sepsis associated with the condition, if not acted promptly. Though being unfortunate, we feel greatly relieved that timely intervention by the team of doctors has prevented any further catastrophe to take place.* (The patient's mother)

## 6. Discussion

Isolated fallopian tube torsion has been described as being a rare cause of lower abdominal pain in women. [1] It was first reported in 1890 by Bland-Sutton, and its exact incidence is not known till now, most probably due to its rare nature and under reporting of cases. As per the current data, its approximate incidence is about 1 : 500000 [2]. Isolated torsion is actually defined by torsion of only the fallopian tube without any ovarian involvement. However, it can be predisposed by several factors which include hydrosalpinx and ovarian or parovarian cysts [3]. Youssef et al. noted factors that could possibly influence the occurrence of fallopian tube torsion and divided them into two types: internal and external [4]. Taken together, the existing reports indicate that the mechanism underlying tubal torsion is apparently a sequential mechanical event [5]. It rarely occurs before menarche or during menopause [6]. The lack of specificity of clinical signs and symptoms and the numerous pathologic findings in the pelvis and lower abdomen often fail to alert the physician to the condition, making diagnosis difficult [7]. Regarding diagnosis, finding of high impedance or absence of flow in a tubular structure, especially in a patient with a history of tubal ligation, can be indicative of the diagnosis. [8]. It was initially thought that the condition occurs more often on the right hand side; however, a recent report published a series of 6 cases and was unable to explain the predominance of left side [9]. The definitive diagnosis of tubal torsion is still made retrospectively, usually after diagnostic laparoscopy. At the present time, laparoscopic detorsion of the tube is the preferred treatment unless the tube is gangrenous or malignancy is suspected. Since the patients in majority belong to the reproductive age group, attempt should be made to preserve the tube [10]. Though it is a rare disease, yet it should be an important differential diagnosis in case of a lady presenting with lower abdominal pain with normal ovaries. The diagnosis of isolated fallopian tube torsion by ultrasound is quite challenging. There should always be high suspicion about this condition when there is a normal appearing ovary in the presence of ultrasound features of torsion [11]. Isolated fallopian tube torsion is more predominant in middle aged women who are actively involved in athletic activities [12]. The management technique is still controversial. Salpingectomy is the most commonly applied method of management but the recent trends are showing a difference [13]. Isolated fallopian tube torsion is still underdiagnosed. On ultrasound, if the ovaries are found morphologically normal, then, a higher degree of suspicion must be followed for isolated fallopian tube torsion. [14]
